# Contraction mode itself does not determine the level of mTORC1 activity in rat skeletal muscle

**DOI:** 10.14814/phy2.12976

**Published:** 2016-09-28

**Authors:** Satoru Ato, Yuhei Makanae, Kohei Kido, Satoshi Fujita

**Affiliations:** ^1^ Faculty of Sport and Health ScienceRitsumeikan UniversityKusatsuJapan

**Keywords:** Mammalian target of rapamycin complex 1, muscle contraction mode, resistance exercise

## Abstract

Resistance training with eccentric contraction has been shown to augment muscle hypertrophy more than other contraction modes do (i.e., concentric and isometric contraction). However, the molecular mechanisms involved remain unclear. The purpose of this study was to investigate the effect of muscle contraction mode on mammalian target of rapamycin complex 1 (mTORC1) signaling using a standardized force‐time integral (load (weight) × contraction time). Male Sprague–Dawley rats were randomly assigned to three groups: eccentric contraction, concentric contraction, and isometric contraction. The right gastrocnemius muscle was exercised via percutaneous electrical stimulation‐induced maximal contraction. In experiment 1, different modes of muscle contraction were exerted using the same number of reps in all groups, while in experiment 2, muscle contractions were exerted using a standardized force‐time integral. Muscle samples were obtained immediately and 3 h after exercise. Phosphorylation of molecules associated with mTORC1 activity was assessed using western blot analysis. In experiment 1, the force‐time integral was significantly different among contraction modes with a higher force‐time integral for eccentric contraction compared to that for other contraction modes (*P* < 0.05). In addition, the force‐time integral was higher for concentric contraction compared to that for isometric contraction (*P* < 0.05). Similarly, p70S6K phosphorylation level was higher for eccentric contraction than for other modes of contraction (*P* < 0.05), and concentric contraction was higher than isometric contraction (*P* < 0.05) 3 h after exercise. In experiment 2, under the same force‐time integral, p70S6K (Thr389) and 4E‐BP1 phosphorylation levels were similar among contraction modes 3 h after exercise. Our results suggest that mTORC1 activity is not determined by differences in muscle contraction mode itself. Instead, mTORC1 activity is determined by differences in the force‐time integral during muscle contraction.

## Introduction

Skeletal muscle is an essential tissue for motion. The magnitude of the muscle power output is dependent on skeletal muscle mass (Bamman et al. 2000). Therefore, skeletal muscle mass is an important factor augmenting the exertion of muscle power. Skeletal muscle mass is regulated by the net balance of muscle protein synthesis and muscle protein breakdown. An acute bout of resistance exercise increases the amount of muscle protein synthesis to a greater extent than muscle protein breakdown does. Thereby, the net balance of protein metabolism improves significantly for up to 48 h after exercise (Phillips et al. 1997). Repeated bouts of exercise can induce muscle protein accumulation, and subsequently hypertrophy (Adams and Haddad 1996).

Mammalian target of rapamycin complex 1 (mTORC1) is a kinase complex that consists of mTOR, PRAS40, GβL, Raptor, and Deptor. Activation of mTORC1 increases ribosomal protein S6 kinase beta‐1 (p70S6K) and/or eukaryotic translation initiation factor 4E‐binding protein 1 (4E‐BP1) phosphorylation, which is the addition of phosphate to specific amino acid residue of protein kinase and activation with no effect on protein abundance (Bolster et al. 2003; Kubica et al. 2005). Phosphorylated p70S6K upregulates ribosomal protein S6 activity that is a component of the 40S ribosomal subunit (Chung et al. 1992). Phosphorylation of 4E‐BP1 leads to eukaryotic initiation factor 4F formation (eIF4F), which binds to the 5ʹ cap of the mRNA and recruiting 40S ribosomal subunit. This series of processes increases mRNA translation, and subsequently protein synthesis (Gingras et al. 1999). A change in the phosphorylation level of p70S6K and 4E‐BP1 is highly correlated with the muscle protein synthesis rate after resistance exercise (Burd et al. 2010a,b). Thus, resistance exercise‐induced increases in muscle protein synthesis can be predicted by p70S6K and 4E‐BP1 phosphorylation levels. Recent investigations have suggested that extracellular signal‐regulated kinase 1/2 (ERK1/2) of mitogen‐activated protein kinase (MAPK) signaling activates mTORC1 via tuberous sclerosis complex 2 (TSC2) inhibition or regulatory‐associated protein of mTOR (Raptor) phosphorylation (Ma et al. 2007; Miyazaki et al. 2011; Frey et al. 2013). In addition, protein kinase B (Akt) also regulates mTORC1 activity through mTOR and several kinases (Inoki et al. 2002; Chiang and Abraham 2005). Previous studies have reported that ERK1/2 and Akt are phosphorylated by resistance exercise and mechanical loading (Nader and Esser 2001; Bolster et al. 2003; Miyazaki et al. 2011); thus, these kinases contribute to mechanical stress‐induced mTORC1 activation (Hornberger et al. 2004; You et al. 2012). A detailed review on mTORC1 activation and its relation to the regulation of muscle protein metabolism is published elsewhere (Goodman 2014).

It is well known that muscle contraction is carried out by eccentric contraction (lengthening), concentric contraction (shortening), or isometric contraction. Previous studies have shown that eccentric contraction increases mTORC1 activation to a greater extent than other contraction modes does. Animal experiments have also demonstrated that eccentric contraction induces higher p70S6K phosphorylation levels than concentric and isometric contraction does (Nader and Esser 2001; Burry et al. 2007). In addition, human studies have reported that p70S6K phosphorylation is higher in eccentric contraction than in concentric contraction after resistance exercise (Eliasson et al. 2006; Rahbek et al. 2014). Together, these studies have shown that eccentric contraction consistently induces higher mTORC1 activation compared to that induced by other contraction modes.

It is well known that the force‐time integral (area under the real‐time contact force curve, or load (weight) × contraction time) also affects the magnitude of mTORC1 activation. Terzis et al. (2010) have reported that the level of mTORC1 activation relates to the number of sets during resistance exercise in humans. Similarly, three sets of resistance exercises produce higher p70S6K phosphorylation and subsequent muscle protein synthesis than does one set of resistance exercise (Burd et al. 2010a). Resistance exercise‐induced increases in muscle protein synthesis also depend on exercise intensity (repetition maximum) (Kumar et al. 2009). However, exercise at 30% of a 1 repetition maximum (1RM) compared with 90% of a 1RM has been shown to exert a larger exercise volume (force‐time integral), a greater increase in p70S6K phosphorylation level, and greater muscle protein synthesis than does 90% at a 1RM in the human study (Burd et al. 2010b). These findings indicate that exercise volume load is a critical determining factor in mTORC1 activity and muscle protein synthesis levels after resistance exercise. Previous studies assessing the effect of different contraction modes on mTORC1 activity using similar contraction times (same total number of reps) have shown that eccentric contraction also induces higher exercise volume loads compared with other contraction modes (Eliasson et al. 2006; Rahbek et al. 2014). Hence, eccentric contraction‐induced higher mTORC1 activation may also be triggered by the magnitude of the exercise volume load.

Previous experiments have suggested that divergence in muscle contraction modes also differentially affects mTORC1 upstream regulators such as ERK1/2 and Akt. Franchi et al. (2014) have reported that ERK1/2 activation is higher in eccentric contraction compared to concentric contraction in humans. Additionally, animal studies have shown that eccentric contraction induces higher Akt phosphorylation compared to concentric contraction (Nader and Esser 2001). Therefore, contraction mode‐induced differences in mTORC1 activity may be regulated by a difference in mTORC1 upstream‐regulator activation. However, there are no current studies assessing the effect of different contraction modes on mTORC1 and upstream‐regulator activity during resistance exercise with a matched force‐time integral. Additionally, the mechanism of the effect of different muscle contraction modes on the level of mTORC1 activity is poorly understood. Therefore, the purpose of this study was to investigate the effect of different contraction modes on mTORC1 signaling with consideration of force‐time integral using an in vivo model.

## Materials and Methods

### Animals and experimental procedure

The study protocol was approved by the Ethics Committee for Animal Experiments at Ritsumeikan University, Japan. Sixty Sprague–Dawley rats, aged 10 weeks, were purchased from CREA Japan (Tokyo, Japan). All animals were housed for 1 week in an environment maintained at 22–24°C with a 12 h–12 h light–dark cycle and received food and water ad libitum. The rats were randomly assigned to three experimental groups (*n* = 5/group/time point) designated as: eccentric contraction (EC; weight, 371.7 ± 8.8 g), concentric contraction (CC; weight, 378.1 ± 8.4 g), and isometric contraction (IC; weight, 380.7 ± 8.3 g). Subsequently, the rats were exercised after a 12 h overnight fast. The rats were killed immediately or 3 h after an acute bout of exercise, and the gastrocnemius muscle was removed immediately (within 5 min). Time points for muscle sampling were determined based on those used in previous studies. Sampling immediately after exercise was chosen in order to assess the upstream‐regulatory factors of mTORC1 activity, while sampling 3 h after exercise was selected in order to evaluate the activation of mTORC1 (Nader and Esser 2001; Ogasawara et al. 2014). After the wet weight of the muscle was measured (Table 1), the tissues were rapidly frozen in liquid nitrogen and stored at −80°C until analysis.

**Table 1:** Animal characteristics

	CON	EC	CC	IC
Body weight, g		371.7 ± 8.8	378.1 ± 8.4	380.7 ± 8.3
Gastrocnemius muscle wet weight, g				
Experiment 1				
0 h	2.05 ± 0.02	2.18 ± 0.03^a^	2.36 ± 0.03^a^, ^c^, ^b^	2.21 ± 0.04^a^
3 h	2.1 ± 0.03	2.18 ± 0.03	2.24 ± 0.04	1.96 ± 0.04
Experiment 2				
0 h	2.08 ± 0.02	2.37 ± 0.06^a^	2.39 ± 0.06^a^	2.38 ± 0.1^a^
3 h	1.97 ± 0.08	2.04 ± 0.08	2.16 ± 0.09	1.99 ± 0.03

Values are means ± SE.

CON, control muscle; EC, eccentric contraction; CC, concentric contraction; IC, isometric contraction.

a *P *<* *0.05 vs. CON.

b *P *<* *0.05 vs. EC.

c *P *<* *0.05 vs. CC.

### Resistance exercise protocols

Under inhaled isoflurane anesthesia (with 2% concentration used for maintenance of anesthesia; Shinano Seisakusho, Tokyo, Japan), the right lower leg of each rat was shaved and cleaned with alcohol wipes. The rats were then positioned with their right foot on a footplate in the prone posture. The triceps surae muscle was stimulated percutaneously with disposable electrodes (Vitrode V, Ag/AgCl; Nihon Kohden, Tokyo, Japan), which were cut to measure 10 mm × 5 mm and connected to an electrical stimulator and an isolator. The right gastrocnemius muscle was exercised (3 sec stimulation × 10 contractions, with a 7‐sec interval between contractions, per set, with 3‐min rest intervals). The nonexercised left gastrocnemius muscle served as the internal control (CON). We previously reported that the percutaneous electrical stimulation of the gastrocnemius muscle increases mTORC1 activation with concomitant increase in muscle protein synthesis (Ogasawara et al. 2014; Kido et al. 2016). Moreover, 18 exercise sessions induced muscle hypertrophy (Ogasawara et al. 2013a). Therefore, we choose to study the gastrocnemius muscle in this study. The voltage (~30 V) and stimulation frequency (100 Hz) were adjusted to produce maximal isometric tension. The differential contraction mode was achieved by changing the ankle joint angle (IC, at 90°; EC, 60°–105°; CC, 105°–60°; joint angular velocity was set at 15°/sec in both EC and CC groups). Output torque was collected (torque meter: Unipulse Corporation, Tokyo, Japan; and A/D converter: CONTEC, Tokyo, Japan) continuously with a sampling rate of 500 Hz and analyzed using Microsoft Excel 2011. The mean force‐time integral was calculated using 50,000 points (500 Hz × 100 sec) of output torque integration per set. Subsequently, values were integrated for each set. First, as a pilot study, all groups were exercised using the same exercise time (five sets) to confirm that our instruments worked similarly as in previous studies (Experiment 1). Subsequently, the groups were exercised using a matched force‐time integral (Experiment 2). The number of sets was modified among the contraction modes (EC, three sets; CC, four sets; IC, five sets) in order to standardize the groups to an equivalent force‐time integral to five sets of isometric contraction (~4100 Nm/sec).

### Western blot analysis

Western blot analysis was performed as reported previously (Kido et al. 2016). Briefly, muscle samples were homogenized in a homogenization buffer containing 100 mmol/L Tris·HCl (pH 7.8), 1% NP‐40, 0.1% SDS, 0.1% sodium deoxycholate, 1 mmol/L EDTA, and 150 mmol/L NaCl, with a protease and phosphatase inhibitor cocktail (Thermo Fisher Scientific, Waltham, MA) using a pestle homogenizer. Homogenates were centrifuged at 10,000 *g* for 10 min at 4°C. The supernatant was removed, and the protein concentration for each sample was determined using the Protein Assay Rapid kit (Wako, Osaka, Japan). The samples were diluted in a 3× sample buffer (1.0% vol/vol β‐mercaptoethanol, 4.0% wt/vol SDS, 0.16 mol/L Tris·HCl, pH 6.8, 43% vol/vol glycerol, and 0.2% wt/vol bromophenol blue) and boiled at 95°C for 5 min. Using 5–20% SDS‐polyacrylamide gels, 20 μg of protein was separated by electrophoresis and subsequently transferred to polyvinylidene difluoride membranes. After the transfer, the membranes were washed in Tris‐buffered saline containing 0.1% Tween 20 (TBST), and membranes were then blocked with 5% skim milk in TBST for 1 h at room temperature. After blocking, the membranes were washed and incubated overnight at 4°C with primary antibodies, including p‐ERK1/2, total‐ERK1/2, p‐Akt (Thr308), p‐Akt (Ser473), total Akt, p‐mTOR (Ser2448), total‐mTOR, p‐p70S6K (Thr389), total p70S6K, p‐Ribosomal protein S6 (rpS6; Ser240/244), total ribosomal protein S6 (Cell Signaling Technology, Danvers, MA), p‐TSC2 (Ser664; Abcam, Cambridge, UK), and total TSC2 (Cell Signaling Technology). The membranes were then washed again in TBST and incubated for 1 h at room temperature with the appropriate secondary antibodies. Chemiluminescent reagents (Luminata Forte Western HRP substrate; Merck Millipore, Darmstadt, Germany) were used to facilitate the detection of protein bands. Images were scanned using a chemiluminescence detector (ImageQuant LAS 4000; GE Healthcare, Buckinghamshire, UK). Band intensities were quantified using ImageJ 1.50f (National Institute of Health, Bethesda, MD). A standardized internal control sample mixture was used for gel‐to‐gel validation. The percentage of variation in the standardized internal control between gels was approximately 8%. Phosphorylation levels were determined by the expression of phosphorylated protein divided by expression of nonphosphorylated total protein.

### Statistical analysis

A one‐way analysis of variance was used to evaluate changes in the phosphorylation of signaling molecules and mechanical parameters. Post hoc analyses were performed using *t*‐tests, with a Benjamini and Hochberg false discovery rate correction for multiple comparisons when appropriate (JMP 10.0; SAS Cary, NC). All values are expressed as means ± SE. The level of significance was set at *P *<* *0.05.

## Results

### Force production characteristics in different contraction modes

Peak torque was significantly different among the three experimental groups, with that in the EC group higher than that in the other groups (*P* < 0.05 vs. CC, *P* < 0.05 vs. IC), and that in the CC group higher than that in the IC group (Table 2).

**Table 2:** Force production characteristics in different contraction modes

	EC	CC	IC
Peak torque, Nm	0.35 ± 0.01^a^, ^b^	0.26 ± 0.01	0.22 ± 0.01

Values are means ± SE.

EC, eccentric contraction; CC, concentric contraction; IC, isometric contraction.

a *P *<* *0.05 vs. CC.

b *P *<* *0.05 vs. IC.

### Experiment 1: different contraction modes with similar exercise times

#### Force‐time integral

**Figure 1:**
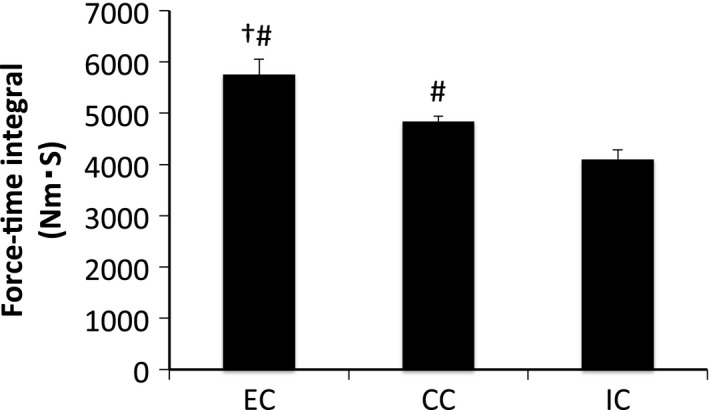
Force‐time integral in different modes of muscle contraction. EC, eccentric contraction; CC, concentric contraction; IC, isometric contraction. Values are means ± SE. ^†^ *P* < 0.05 vs. CC; ^#^ *P* < 0.05 vs. IC.

The mean force‐time integral (exercise volume load) was calculated across the five sets of muscle contraction (Fig. 1). The mean force‐time integral was significantly different among experimental groups, with that in the EC group higher compared with that in the other groups (*P* < 0.05 vs. CC, *P* < 0.05 vs. IC). In addition, the CC group had a higher force‐time integral value compared with that for the IC group (*P* < 0.05).

#### Upstream‐regulatory factors of mTORC1

**Figure 2:**
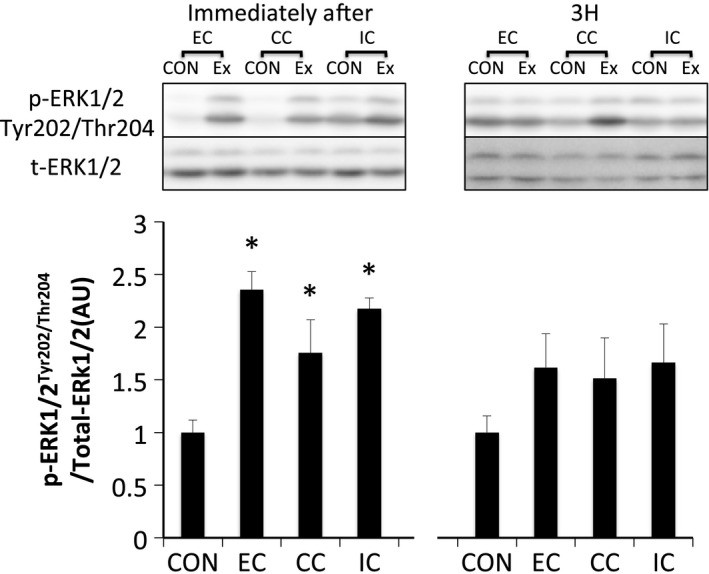
Phosphorylated ERK1/2 Thr202/Tyr204 relative to total protein content after different modes of muscle contraction. EC, eccentric contraction; CC, concentric contraction; IC, isometric contraction; CON, control muscle; Ex, exercised muscle. Values are means ± SE. *P < 0.05 vs. CON; AU, arbitrary unit.

**Figure 3:**
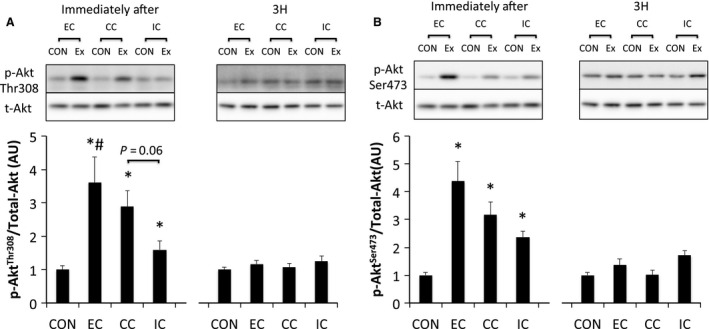
Phosphorylated Akt Thr308 (A) and Ser473 (B) relative to total protein content after different modes of muscle contraction. EC, eccentric contraction; CC, concentric contraction; IC, isometric contraction; CON, control muscle; Ex, exercised muscle. Values are means ± SE. *P < 0.05 vs. CON; ^#^ *P* < 0.05 vs. IC; AU, arbitrary unit.

ERK1/2^Thr202/Tyr204^ phosphorylation was significantly elevated immediately after exercise in all groups (*P* < 0.05 vs. CON). However, the level of ERK1/2 phosphorylation was similar among all groups. Phosphorylation of ERK1/2 returned to the level in the CON at 3 h after exercise (Fig. 2). Akt^Thr308^ phosphorylation was significantly elevated immediately after exercise in all groups (*P* < 0.05 vs. CON) and was higher in the EC group than in the IC group (*P* < 0.05). The level of phosphorylated Akt^Thr308^ was reduced to the level of the CON at 3 h after exercise (Fig. 3A). Phosphorylation of Akt^Ser473^ was significantly increased immediately after exercise in all groups (*P* < 0.05); however, no significant differences were observed among contraction modes. The level of phosphorylated Akt^Ser473^ returned to the level of the CON at 3 h after exercise (Fig. 3B).

#### Downstream of mTORC1

**Figure 4:**
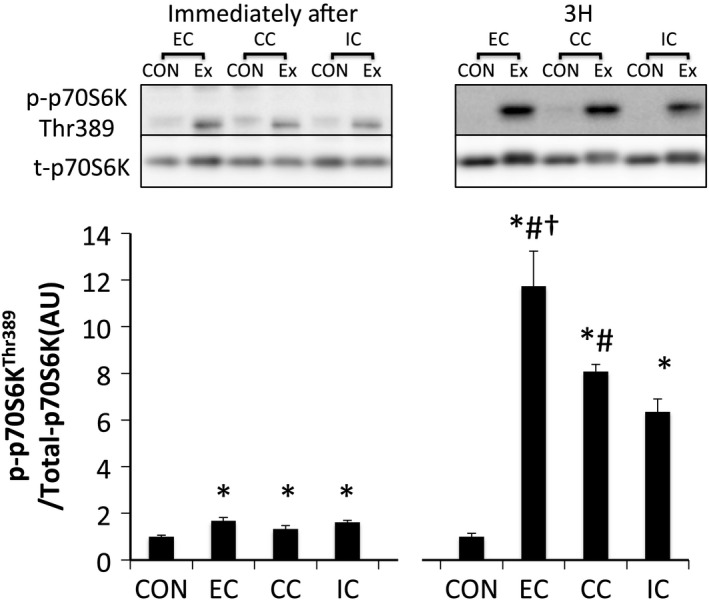
Phosphorylated p70S6K Thr389 relative to total protein content after different modes of muscle contraction. EC, eccentric contraction; CC, concentric contraction; IC, isometric contraction; CON, control muscle; Ex, exercised muscle. Values are means ± SE. *P < 0.05 vs. CON; ^†^ *P *<* *0.05 vs. CC; ^#^ *P *<* *0.05 vs. IC; AU, arbitrary unit.

**Figure 5:**
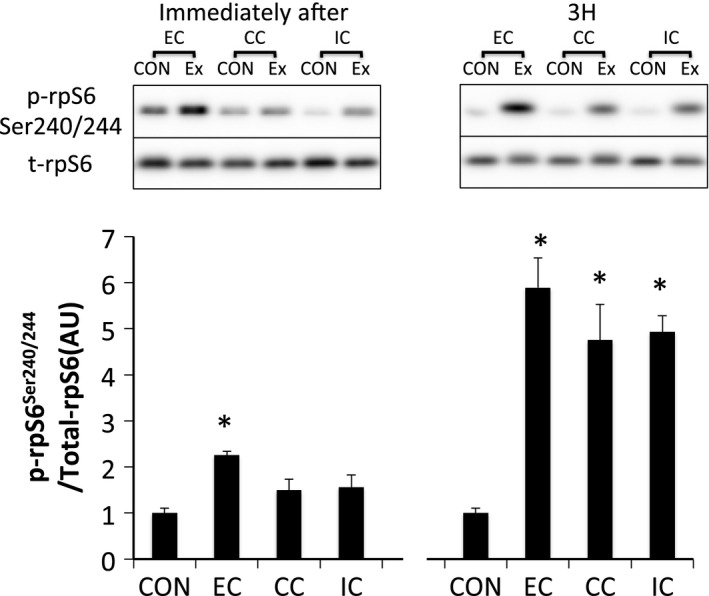
Phosphorylated rpS6 Ser240/244 relative to total protein content after different modes of muscle contraction. EC, eccentric contraction; CC, concentric contraction; IC, isometric contraction; CON, control muscle; Ex, exercised muscle. Values are means ± SE. *P <* *0.05 vs. CON; AU, arbitrary unit.

Phosphorylation of p70S6K^Thr389^ was significantly increased immediately and 3 h after exercise in all groups without any significant group differences (*P* < 0.05 vs. CON) (Fig. 4). Furthermore, the magnitude of phosphorylation was significantly different among contraction modes at 3 h after exercise. The magnitude of phosphorylation in the EC group was higher than that of the other contraction modes (*P* < 0.05 vs. CC, *P* < 0.05 vs. IC), and the CC group's magnitude was higher compared with the IC group at 3 h after exercise (*P* < 0.05) (Fig. 4). Immediately after exercise, ribosomal protein S6^Ser240/244^ was significantly phosphorylated only in the EC group (*P* < 0.05 vs. CON). Subsequently, the phosphorylation of ribosomal protein S6 (rpS6)^Ser240/244^ was significantly increased 3 h after exercise in all groups (*P* < 0.05 vs. CON) (Fig. 5).

### Experiment 2: different contraction modes with equivalent force‐time integral

#### Force‐time integral

**Figure 6:**
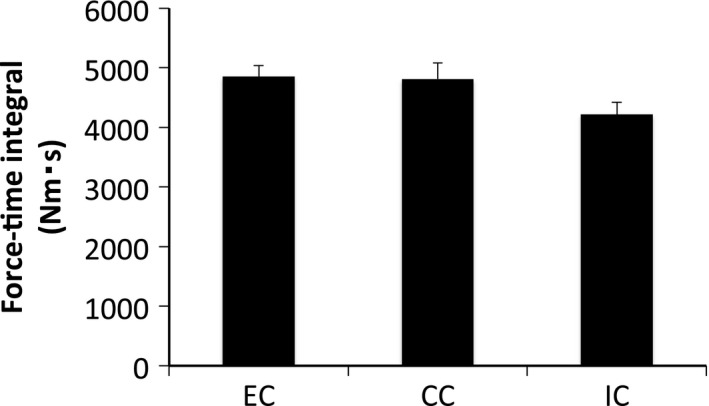
Force‐time integral after different modes of muscle contraction. Values are means ± SE.

As intended, there was no statistically significant difference in the mean force‐time integral among the three groups (Fig. 6).

#### Upstream‐regulatory factors of mTORC1

**Figure 7:**
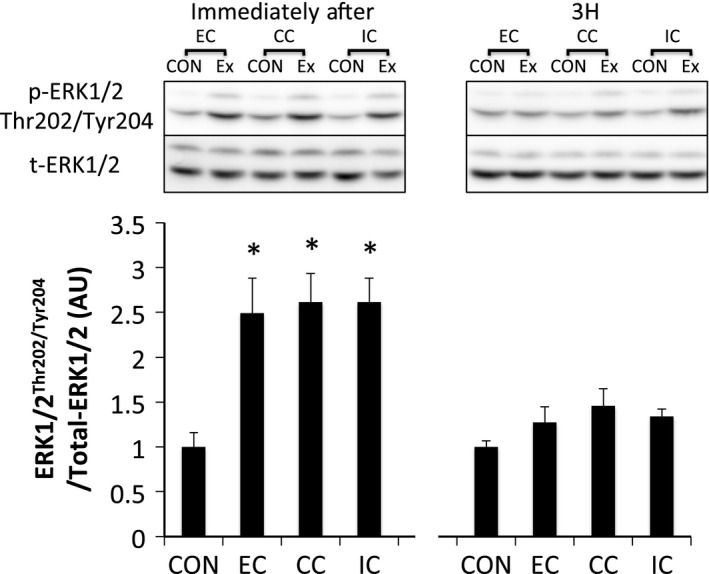
Phosphorylated ERK1/2 Thr202/Tyr204 relative to total protein content after different modes of muscle contraction. EC, eccentric contraction; CC, concentric contraction; IC, isometric contraction; CON, control muscle; Ex, exercised muscle. Values are means ± SE. *P <* *0.05 vs. CON; AU, arbitrary unit.

**Figure 8:**
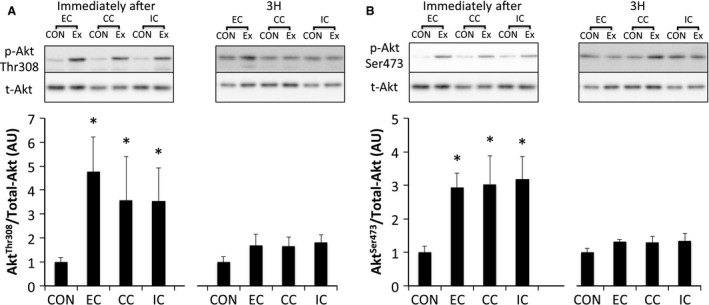
Phosphorylated Akt Thr308 (A), Ser473 (B) relative to total protein content after different modes of muscle contraction. EC, eccentric contraction; CC, concentric contraction; IC, isometric contraction; CON, control muscle; Ex, exercised muscle. Values are means ± SE. *P <* *0.05 vs. CON; AU, arbitrary unit.

**Figure 9:**
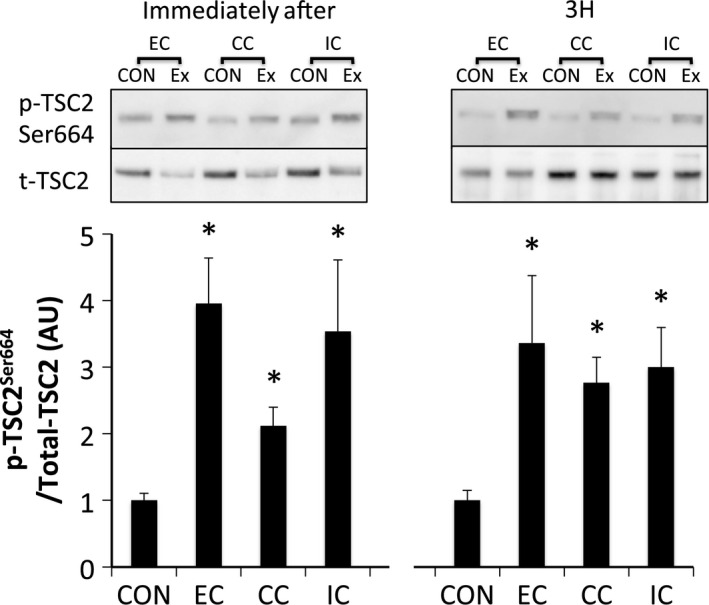
Phosphorylated TSC2 Ser664 relative to total protein content after different modes of muscle contraction. EC, eccentric contraction; CC, concentric contraction; IC, isometric contraction; CON, control muscle; Ex, exercised muscle. Values are means ± SE. *P <* *0.05 vs. CON; AU, arbitrary unit.

**Figure 10:**
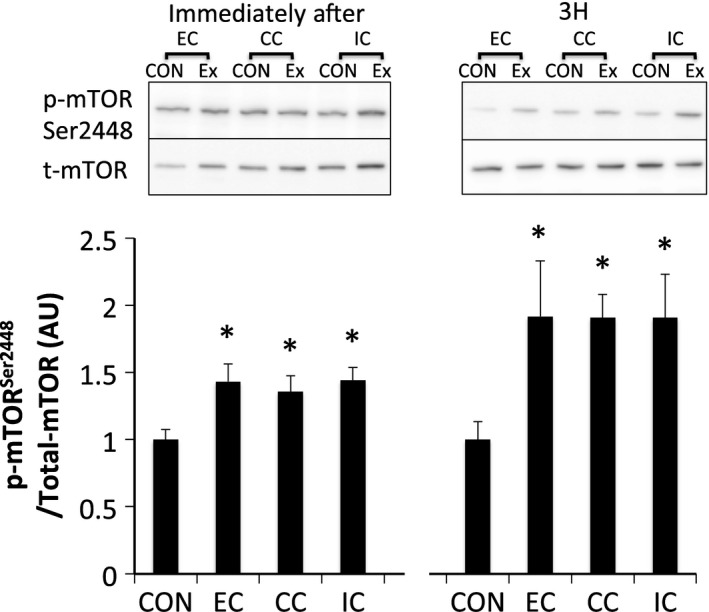
Phosphorylated mTOR Ser2448 relative to total protein content after different modes of muscle contraction. EC, eccentric contraction; CC, concentric contraction; IC, isometric contraction; CON, control muscle; Ex, exercised muscle. Values are means ± SE. *P <* *0.05 vs. CON; AU, arbitrary unit.

Phosphorylated ERK1/2^Thr202/Tyr204^ was significantly increased immediately after exercise (*P* < 0.05 vs. CON). Phosphorylation of ERK1/2 returned to the level of that in the CON by 3 h after exercise (Fig. 7). Phosphorylated Akt^Thr308^ (Fig. 8A) and Akt^Ser473^ (Fig. 8B) were significantly phosphorylated immediately after exercise in all groups (*P* < 0.05 vs. CON). The levels of Akt^Thr308^ and Akt^Ser473^ phosphorylation returned to the levels of those in the CON at 3 h after exercise. Phosphorylation of TSC2^Ser664^ (Fig. 9) and mTOR^Ser2448^ (Fig. 10) were significantly increased immediately and 3 h after exercise in all groups without any significant group differences (*P* < 0.05 vs. CON).

#### Downstream of mTORC1

**Figure 11:**
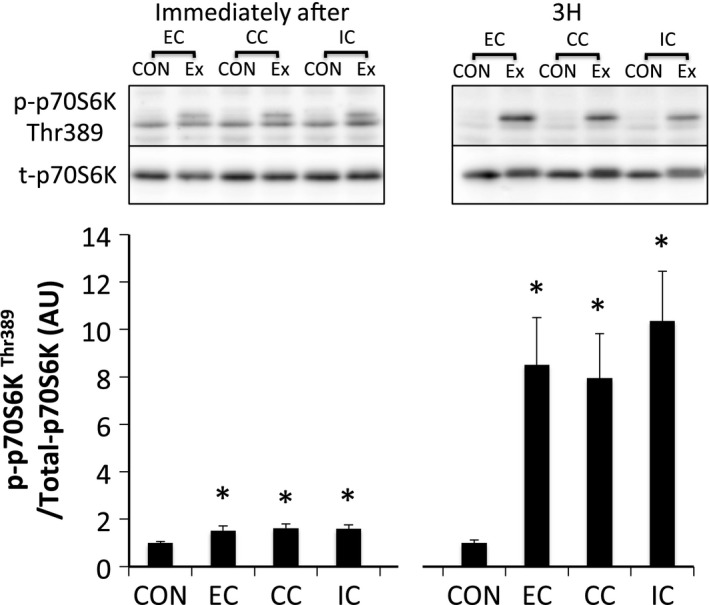
Phosphorylated p70S6K Thr389 relative to total protein content after different modes of muscle contraction. EC, eccentric contraction; CC, concentric contraction; IC, isometric contraction; CON, control muscle; Ex, exercised muscle. Values are means ± SE. *P <* *0.05 vs. CON; AU, arbitrary unit.

**Figure 12:**
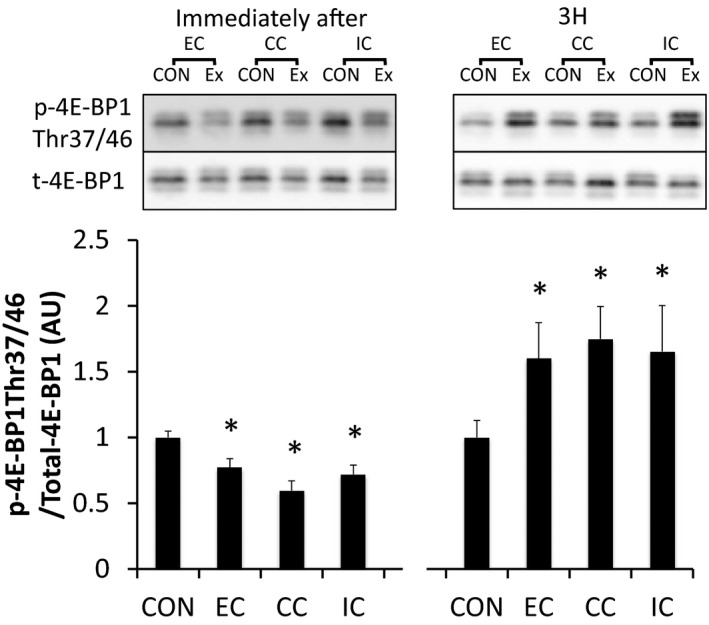
Phosphorylated 4E‐BP1 Thr37/46 relative to total protein content after different modes of muscle contraction. EC, eccentric contraction; CC, concentric contraction; IC, isometric contraction; CON, control muscle; Ex, exercised muscle. Values are means ± SE. *P <* *0.05 vs. CON; AU, arbitrary unit.

**Figure 13:**
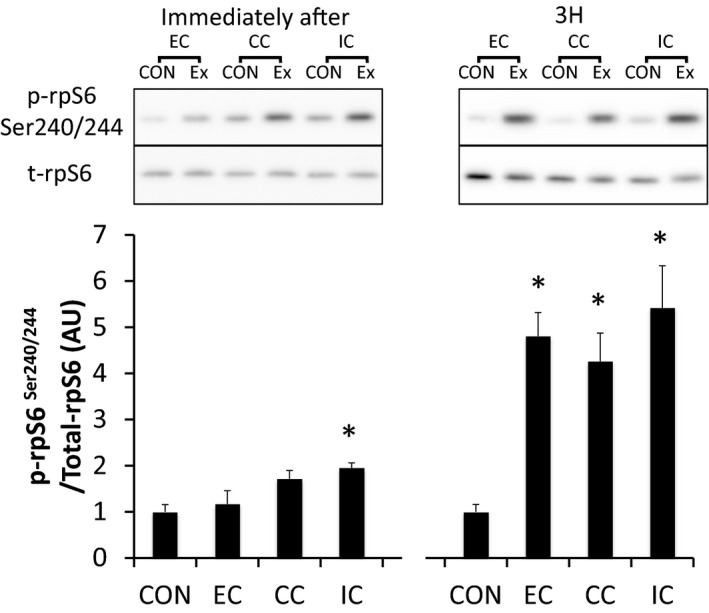
Phosphorylated rpS6 Ser240/244 relative to total protein content after different modes of muscle contraction. EC, eccentric contraction; CC, concentric contraction; IC, isometric contraction; CON, control muscle; Ex, exercised muscle. Values are means ± SE. *P <* *0.05 vs. CON; AU, arbitrary unit.

p70S6K^Thr389^ was significantly phosphorylated immediately and 3 h after exercise in all groups (*P* < 0.05 vs. CON), but was not statistically different among the groups (Fig. 11). Although 4E‐BP1 was dephosphorylated immediately after exercise in all groups (*P* < 0.05 vs. CON), the phosphorylation level was increased 3 h after exercise in all groups (*P* < 0.05 vs. CON) (Fig. 12). Immediately after exercise, level of phosphorylated rpS6^Ser240/244^ was significantly increased only in the IC group (*P* < 0.05 vs. CON). Nevertheless, rpS6 phosphorylation was observed in all groups at 3 h after exercise (*P* < 0.05 vs. CON) with no significant differences among the groups (Fig. 13).

## Discussion

We sought to investigate the mechanism by which different contraction modes affect mTORC1 activity using a matched force‐time integral. In experiment 1, a difference in the contraction mode was found to affect the force‐time integral (EC>CC>IC) when the same number of contractions were applied. The phosphorylation level of p70S6K was also different among the contraction modes with EC>CC>IC. In addition, Akt phosphorylation was higher for EC compared to IC. In contrast, the phosphorylation level of ERK1/2 and Akt were similar among the groups in experiment 2, during which the force‐time integral was adjusted to be equal among the experimental groups. TSC2 and mTOR, downstream of ERK1/2 and Akt, p70S6K and 4E‐BP1 were also phosphorylated similarly among the groups in experiment 2. Therefore, difference in the contraction mode did not affect the magnitude of mTORC1 activity or upstream‐regulator activation levels when the force‐time integral was matched.

In this study, we assessed the effect of differences in contraction mode on the activity of mTORC1 and upstream regulators with consideration for the force‐time integral. In order to define the force‐time integral, we modified a previously used percutaneous electrical muscle contraction experimental model for animals that had been built with the capacity to execute the three different contraction modes (Ogasawara et al. 2013b; Kido et al. 2016). With voluntary contraction, force production is controlled by neural activity, and a difference in contraction modes affects the muscle activation level (Tesch et al. 1990; Babault et al. 2001; Beltman et al. 2004). Accordingly, precise standardization of the muscle activation level is difficult with voluntary contraction in humans, even with a standardized force‐time integral during exercise. On the other hand, electrical stimulation‐induced maximal muscle contraction is suitable for recruiting similar neural activation regardless of muscle contraction mode (Nakazato et al. 2010). We applied an electrical stimulation‐induced maximal muscle contraction with matched repetitions in experiment 1. As expected, eccentric contraction exerted a higher force‐time integral value compared to other contraction modes, consistent with previous studies (Franchi et al. 2014; Rahbek et al. 2014). Based on these results, the total number of sets for the concentric and eccentric contraction was adjusted in order to equalize the force‐time integral among the three types of contraction modes for experiment 2.

Mammalian target of rapamycin complex 1 activity is regulated by several molecular signaling pathways. ERK1/2, a MAPK, is an upstream regulator of mTORC1 activity through the phosphorylation of TSC2 and Raptor. In this study, the phosphorylation level of ERK1/2 was similar among the three groups in both experiments 1 and 2. The phosphorylation level of TSC2 Ser664 (downstream of ERK1/2) was found to be similar among the three groups in experiment 2, while the force‐time integral was the same between groups. In contrast, a previous study reported that eccentric contraction increases ERK1/2 phosphorylation to a greater degree compared to other contraction modes; however, the total contraction time of that study was less than that in this study (total contraction time, ~1.5 sec vs. 150 sec) (Martineau and Gardiner 2001). ERK1/2 phosphorylation has been shown to relate to the force‐time integral but subsequently saturates (Aronson et al. 1997; Martineau and Gardiner 2001). Therefore, ERK1/2 activity may have plateaued in this study.

Akt, another upstream regulator of mTORC1, activates mTORC1 by the phosphorylation of mTOR Ser2448 and PRAS40 (Chiang and Abraham 2005; Wang et al. 2012). A previous study using an animal model suggested that differences in contraction mode may influence the Akt phosphorylation level (Nader and Esser 2001). However, in that study, concentric and eccentric contractions were given by co‐contracting the tibialis anterior and the antagonistic soleus simultaneously for an identical contraction time. In experiment 1, the level of Akt phosphorylation was higher in the EC group than in the IC group, which reflects a group difference in the force‐time integral. However, the Akt phosphorylation level was similar among contraction modes in experiment 2, when the force‐time integral was matched among groups. Furthermore, the phosphorylation level of mTOR Ser2448, one of the downstream targets of Akt, was not different among contraction modes under an equivalent force‐time integral in experiment 2. Therefore, our results suggest that the level of Akt‐mTOR signaling activation is determined by the magnitude of the force‐time integral and not by a difference in contraction mode. Our findings support Russ's previous work suggesting that Akt activity is increased with the force‐time integral in situ (Russ 2008).

We found that a difference in contraction mode affects the magnitude of p70S6K phosphorylation 3 h after exercise with EC>CC>IC in experiment 1. This result supports previous experiments regarding the effect of different muscle contraction modes on mTORC1 activation (Nader and Esser 2001; Burry et al. 2007). Additionally, we examined the effect of different contraction modes on the level of mTORC1 activity under the condition of a standardized force‐time integral and the magnitude of p70S6K phosphorylation was found to be similar among the three types of contraction mode. The phosphorylation level of 4E‐BP1 (downstream of mTORC1) was also similar among all contraction modes at every examined time point. Together, these results suggest that mTORC1 activity and translation initiation are also similar among the three types of contraction mode under a similar force‐time integral. However, a previous human study reported that eccentric contraction increases muscle protein synthesis to a greater extent compared to concentric contraction, despite standardizing the total work (kJ) used during the voluntary contraction (Moore et al. 2005). Although the reason for this mismatch is unclear, the way in which the muscle contraction was derived may have affected the results. As mentioned previously, the amount of active muscle and pattern of muscle activity differs between eccentric and concentric contraction during maximal voluntary contraction (Babault et al. 2001; Beltman et al. 2004). In this study, muscle activation levels were matched among the contraction modes using an electrical stimulation‐induced maximal muscle contraction. Further studies are warranted to clarify the impact of muscle activation level on subsequent mTORC1 activity.

In summary, the present work demonstrates that differences in mTORC1 activity among contraction modes reflects the force‐time integral as seen in the case of standardized contraction times (experiment 1). Moreover, we found that the level of mTORC1 activity was similar among groups under the same force‐time integral (experiment 2). Therefore, difference in contraction modes is not a direct factor in the regulation of mTORC1 and upstream‐regulator activity. Instead, mTORC1 signaling activity was determined by a difference in the force‐time integral during contraction. In this study, we focused on an acute bout of exercise and assessed the effect of different contraction modes on the regulation of signaling molecules involved in mRNA translation. Our results provide useful data related to choosing the type of exercise intervention for rehabilitation purposes, in which resistance exercise with minimal mechanical stress on joints is preferred for the patient population. However, the longitudinal growth of skeletal muscle also needs other adaptations, including nuclear accretion by mature satellite cells (Fry et al. 2014). Accordingly, future studies need to assess the effect of different muscle contraction modes on prolonged muscle hypertrophy.

## Conflict of interest

None declared.
